# Supplementary material for: Characteristics and outcomes of children, adolescents and young adults with relapsed/refractory non-hodgkin lymphoma undergoing autologous stem cell transplant
Source: BMC Cancer. 2023 Dec 20;23:1258. doi: 10.1186/s12885-023-11712-6 (PMC10734180; doi:10.1186/s12885-023-11712-6)
Supplement: Supplementary file 1 — Additional file 1: Supplementary Table 1. Causes of death. [file 12885_2023_11712_MOESM1_ESM.docx]

Supplementary Table 1. Causes of death

|  | **All (N=60)**  **n (%)** | **Age ≤ 25 years (N=10)**  **n (%)** | **Age > 25 years**  **(N=50)**  **n (%)** |
| --- | --- | --- | --- |
| Progression of lymphoma | 43 (72%) | 6 (60%) | 37 (74%) |
| SPM | 5 (8%) | 0 | 5 (10%) |
| Infection | 3 (5%) | 0 | 3 (6%) |
| Cardiac failure | 1 (2%) | 1 (10%) | 0 |
| Chronic GVHD | 1 (2%) | 1 (10%) | 0 |
| Other/unknown | 7 (12%) | 2 (20%) | 5 (10%) |

SPM = second primary malignancies; GVHD = graft versus host disease.
