# Supplementary material for: Characteristics and outcomes of children, adolescents and young adults with relapsed/refractory non-hodgkin lymphoma undergoing autologous stem cell transplant
Source: BMC Cancer. 2023 Dec 20;23:1258. doi: 10.1186/s12885-023-11712-6 (PMC10734180; doi:10.1186/s12885-023-11712-6)
Supplement: Supplementary file 3 — Additional file 3: Supplementary Table 3. Univariate assessments for overall survival (OS). [file 12885_2023_11712_MOESM3_ESM.docx]

Supplementary Table 3. Univariate assessments for overall survival (OS)

| **Parameter** | **Hazard Ratio (95% CI)** | **p-value** |
| --- | --- | --- |
|  |  |  |
| **Age (years)** |  |  |
| ≤25 | *ref* |  |
| >25 | 1.5 (0.7 – 3.2) | 0.24 |
| **Gender** |  |  |
| Female | *Ref* |  |
| Male | 1.2 (0.7 – 2.1) | 0.58 |
| **Histology** |  |  |
| DLBCL | *ref* |  |
| PMBCL | 0.9 (0.5 – 2.0) | 0.88 |
| T-NHL | 1.1 (0.6 – 2.2) | 0.74 |
| **Response prior to ASCT** |  |  |
| CR | *ref* |  |
| PR | 4.3 (2.2 – 8.2) | **<0.0001** |
| SD | 6.0 (2.0 – 18.6) | **0.002** |
| PD | 7.3 (2.4 – 22.5) | **0.001** |
| **Conditioning regimen** |  |  |
| BEAM +/- rituximab | *ref* |  |
| GemBuMel - based | 1.2 (0.63 – 2.21) | 0.62 |
| **KPS/LPS** |  |  |
| 100 | *ref* |  |
| 90 | 1.0 (0.5 – 2.1) | 0.99 |
| 80 | 1.5 (0.6 – 3.5) | 0.40 |
| 70 | 0.8 (0.1 – 3.4) | 0.80 |
| 60 | 1.8 (0.0 – 13.4) | 0.72 |
| **sIPI** |  |  |
| 0-1 | *ref* |  |
| 2-3 | 0.8 (0.3 – 2.5) | 0.76 |
| **Number of treatment lines prior to ASCT (continuous)** | 1.5 (1.1 – 1.9) | **0.008** |
| **Double or Triple Hit / Double Expressor status ^¥^ (continuous)** | 1.8 (0.7 – 4.7) | 0.26 |

**Abbreviations:** DLBCL = diffuse large B-cell lymphoma; PMBCL = primary mediastinal B-cell lymphoma; T-NHL = T-cell non-hodgkin lymphoma; ASCT = autologous hematopoietic stem cell transplantation; BEAM = BCNU, etoposide, cytarabine and melphalan; GemBuMel = gemcitabine, busulfan and melphalan; KPS/LPS = karnofsky/lansky performance scale; CR = complete remission; PR = partial remission; SD = stable disease; PD = progressive disease; sIPI = secondary international prognostic index;

^¥^ Considered positive if either of the following positive: double hit / triple hit / double expressor. Calculated for patients with DLBCL histology only.
